# Ketosis-Prone Diabetes (KPD): case report

**DOI:** 10.1186/1758-5996-7-S1-A93

**Published:** 2015-11-11

**Authors:** Rafael Buck Giorgi, Andressa Canzian Lopes Lubanco, Bianca Carmona Marmille Takatsu, Aline Barbosa Silva Ribeiro, Ana Leticia de Souza Godoi, Cecilia Nogueira Machado, Natalia Gonçalves Rodrigues, Liane Cristina Borges Vivaldi, Maria Teresa Verrone Quilici, Alexandre Eduardo Franzin Vieira

**Affiliations:** 1 Hospital Regional de Sorocaba, Barueri, Brazil

## Background

The ketosis-prone diabetes syndrome (KPD) is characterized by a severe dysfunction of pancreatic beta cells, leading to the diagnosis of diabetic ketoacidosis (DKA) in patients that do not present the typical phenotype of diabetes mellitus type 1 (DM1). This heterogenic condition has been observed more often in urgent care facilities, leading to confusion in diagnosis and potential treatment errors, with negative outcomes. KPD must be suspected in patients with clinical presentation and laboratory findings of diabetic ketoacidosis that do not fit the clinical and laboratorial profiles of DM1. The incidence rate of KPD is highest in African-Americans and Hispanics, and obesity is the most common phenotype. Overall, patients have type 2 diabetes mellitus (DM2) with considerable family history. Four subgroups were identified for KPD: presence or absence of autoimmune response against beta cells (A+ or A-) and presence or absence of pancreatic reserve (B+ or B-).

## Case report

L.P.L, male, 31, mulatto, obese (body mass index: 31 kg/m^2^), with no previous comorbidities, arrived at the Emergency Health Care Facility of an University Hospital with polyuria, polydipsia, and weight loss in the past 5 days, with worsening of symptoms in the past few h. Capillary glycemic Results were HI at admission. Tests showed glycemic values of 577mg/dL; metabolic acidosis in arterial gasometry and positive ketonemia and ketonuria were observed, confirming DKA. There were no signs of infection; the patient denied using medication or illicit drugs, and stated he had never been tested for diabetes. After KPD was reverted, patient was discharged with low-dose insulin (0.2u/Kg). During etiological investigation, glycated hemoglobin of 6.8% (4-6%) and C peptide of 1.9ng/mL (1.1-4.4) were observed; and tests for autoantibodies were negative. In the outpatient follow-up, insulin was reduced and later discontinued; metformin was prescribed, with good glycemic control.

## Conclusion

KPD is a challenging syndrome for GPs and endocrinologists. Its prevalence has been increasing, but diagnosis allows for fast intervention and accurate treatment. The mechanisms of damage of beta cells are still unknown; therefore, more research is still needed.

